# Surgical outcomes of profunda artery perforator flap in head and neck reconstruction: A systematic review and meta‐analysis

**DOI:** 10.1002/hed.27891

**Published:** 2024-07-30

**Authors:** Filippo Marchi, Andrea Iandelli, Gian Marco Pace, Elisa Bellini, Alessandro Tirrito, Andrea Costantino, Luca Cerri, Antonio Greco, Antonella Polimeni, Giampiero Parrinello, Giorgio Peretti, Armando De Virgilio

**Affiliations:** ^1^ IRCCS Ospedale Policlinico San Martino Genoa Italy; ^2^ Department of Surgical Sciences and Integrated Diagnostics (DISC) University of Genova Genoa Italy; ^3^ Department of Biomedical Sciences Humanitas University Milan Italy; ^4^ Otorhinolaryngology Unit IRCCS Humanitas Research Hospital Milan Italy; ^5^ Department of Otolaryngology – Head and Neck Surgery AdventHealth Orlando Celebration Florida USA; ^6^ Department of Sensory Organs Sapienza University of Rome Rome Italy; ^7^ Department of Odontostomatological and Maxillofacial Sciences Sapienza University of Rome Rome Italy

**Keywords:** head and neck cancer, microsurgery, PAP flap, perforator flap, surgical complication

## Abstract

**Objective:**

This study aims to evaluate the efficacy of the profunda artery perforator (PAP) flap in head and neck reconstruction.

**Methods:**

A single arm meta‐analysis was performed for flap survival rate (primary outcome), reoperation for major complication, and overall complication rates (secondary outcomes).

**Results:**

The search strategy yielded a total of 295 potentially relevant publications, of which 13 were included. A total of 305 patients (males: 80.8%, *n* = 232/281), with a median age of 56.1 years (*n* = 305/305; 95% CI 53.9–63), who underwent a total of 307 PAP flap reconstructions for head and neck defects were included. Flap survival rate was 100% (*n* = 306/307; 95% CI 99.6%–100%), with a reoperation rate for major complications of 3.7% (*n* = 15/307; 95% CI 1.85%–6.1%) and an overall complication rate of 26.5% (*n* = 92/307; 95% CI 15.7%–38.9%). Notable postoperative complications included wound dehiscence (*n* = 15/307, 4.9%), delayed healing (*n* = 14/307, 4.6%), and wound infection (*n* = 12/307, 3.9%). Partial flap necrosis and hematoma occurred in 2.6% of cases (*n* = 8/307), while arterial and venous thrombosis were documented in 0.7% (*n* = 2/307) and 1.3%, respectively (*n* = 4/307).

**Conclusion:**

The application of the PAP flap in head and neck reconstructions showed several favorable aspects, such as an exceptionally low flap failure rate, versatility in achieving variable dimensions, and a relatively low incidence of complications. PAP flap might be considered as a compelling alternative to the traditionally employed soft tissue free flaps in head and neck reconstruction.

## 1 INTRODUCTION

In recent years, free flaps have emerged as the preferred method for head and neck reconstruction. Among these, the anterolateral thigh flap (ALT), the radial forearm flap (RFFF), and the latissimus dorsi (LD) flap have become workhorse flaps for soft‐tissue reconstruction.^1^, ^2^ These flaps may present specific reconstructive requirements, other than various limitations. For example, the ALT flap may display variations in the perforator–pedicle relationship, particularly when dealing with a dominant oblique or transverse branch of the circumflex artery.^3^, ^4^ Additionally, patients undergoing reconstruction with this flap report may experience lower limb weakness and potential dissatisfaction with the aesthetic outcome due to the location of the donor site scar.^5^ The RFFF aside from its potential to induce aesthetically displeasing scarring can result in sensory loss in the volar region of the hand. Moreover, this flap poses significant limitations in terms of size and volume. On the other hand, the LD flap has a considerable thickness, which can be limiting in several head and neck reconstructions, and it may also present intraoperative challenges due to the necessity of changing patient positioning.

Since their introduction in 1989 by Koshima and Soeda, perforator flaps quickly gained popularity for soft‐tissue reconstructions.^6^ The concept of “free‐style free flaps” by Mardini et al. further expanded their use, allowing for dissection based on skin perforators identified with a Doppler, regardless of vessel origin.^7^, ^8^

Among them the profunda artery perforator (PAP) flap, harvested from the posteromedial region of the thigh, has gained popularity as an alternative to DIEP in autologous breast reconstruction,^9^, ^10^ but also in numerous other reconstruction sites.^11^, ^12^ The PAP flap offers several advantages, including low functional morbidity after harvest, adequate flap volume, remarkable versatility, and scarring often perceived by patients as less noticeable due to its location in the posterior gluteal crease. In slim patients, PAP flap offers advantages by providing larger volumes for defect coverage compared to either deep inferior epigastric perforator flaps or ALT perforator flaps. Additionally, since the donor site is easily accessible with the patient in a supine frog‐leg position, there is no need to move the patient during or after flap harvest. This is particularly beneficial during a two‐team surgery, allowing both teams to operate simultaneously without hindering each other.

The role of the PAP flap in the head and neck reconstruction has not been well‐defined in the current literature. This systematic review and meta‐analysis aims to assess the efficacy of the PAP flap in head and neck reconstruction in terms of survival and complications rates, shedding a light on its potential as a promising option in the field.

## 2 MATERIALS AND METHODS

This systematic review and meta‐analysis was reported according to the Preferred Reporting Items for Systematic Reviews and Meta‐Analyses (PRISMA) statement.^13^ No review protocol was registered for this study. Neither ethics approval nor informed consent were required for this study since all reported data were obtained from the available published literature.

### 2.1 Eligibility criteria

Inclusion criteria were established according to the PICOS tool: *Patients* (P), adults with a head and neck surgical defect (e.g., skin, oral cavity, and pharyngeal defects); *Intervention* (I), PAP flap; *Comparator* (C), none; *Outcomes* (O), flap survival rate (primary outcome), reoperation for major complication and overall complication rates (secondary outcomes); *Study design* (S), retrospective and prospective cohort studies. Studies were excluded if they (a) were not in English, (b) were not available in full‐text form, (c) reported insufficient clinical data or data were not extractable, (d) were ongoing projects, (e) included only patients who underwent PAP flap reconstruction for non‐head and neck defects, (f) included less than five PAP flaps, and (g) the article type was either a review, case report, conference abstract, or book chapter.

No publication date restriction was imposed, and articles had to be published in a peer‐reviewed journal.

### 2.2 Data source and study searching

A comprehensive literature search was independently conducted by three authors (L.C., E.B., and A.T.) on the Scopus, PubMed/MEDLINE, Cochrane, and Google Scholar databases. The last research was carried out on November 26, 2023. Appropriate keywords, phrases, and medical subject headings (MeSH) terms were used according to each database requirement. As an example, the following search strategy was used for PubMed/MEDLINE: “PAP flap” OR “Profunda Artery Perforator flap” AND ((“Neoplasms”[Mesh]) OR (“Carcinoma”[Mesh]) OR (“Cancer” [All Fields]) OR (“Tumor” [All Fields])) AND (Head and Neck). Articles cited by all papers selected for full‐text assessment and all retrieved systematic and narrative reviews were examined for further studies retrieval. The “cited by” function on Google Scholar was used for search completion.

### 2.3 Data collection process

Publications identified through databases searching were merged and deduplicated using the reference management software EndNote® X9 (version X9.3.3). The three authors (L.C. and E.B.) separately performed a close reading of all papers deemed relevant by screening the list of titles and abstracts retrieved by the literature search. Disagreements were resolved by referring to two different authors (A.D.V. and F.M.). The most updated and inclusive data for each study were chosen for abstraction. A standardized Excel form was compiled by two authors two authors (L.C. and E.B.) to extract the following characteristics of the included studies: study design, number of patients, patient demographics and body mass index (BMI), number of PAP flaps, site of defects, flap size and characteristics, flap total or partial failure, other complications, and mean operative time. Finally, another author (G.M.P.) checked for accuracy. All events and conditions classified as “complication” by the authors were extracted. This includes any direct or indirect consequences of surgery that impaired the patient's smooth healing and recovery. Given the variation in reporting between different studies, this broad definition captures the range of complications documented in the reviewed literature.

### 2.4 Risk of bias and study quality assessment

The National Institute for Health and Clinical Excellence (NICE) quality assessment tool^14^ was used independently by two authors (G.M.P. and L.C.). A funnel plot was created using the effect size for flap survival rate, reoperation rate for major complication and overall complication rate to examine potential publication bias.^15^

### 2.5 Data synthesis and statistical analysis

Descriptive statistics were used to summarize data from the included studies. Dichotomous variables were reported as counts and percentages, while continuous variables as median and 95% confidence interval (CI) calculated through the method described by McGrath et al.^16^

**FIGURE 1:**
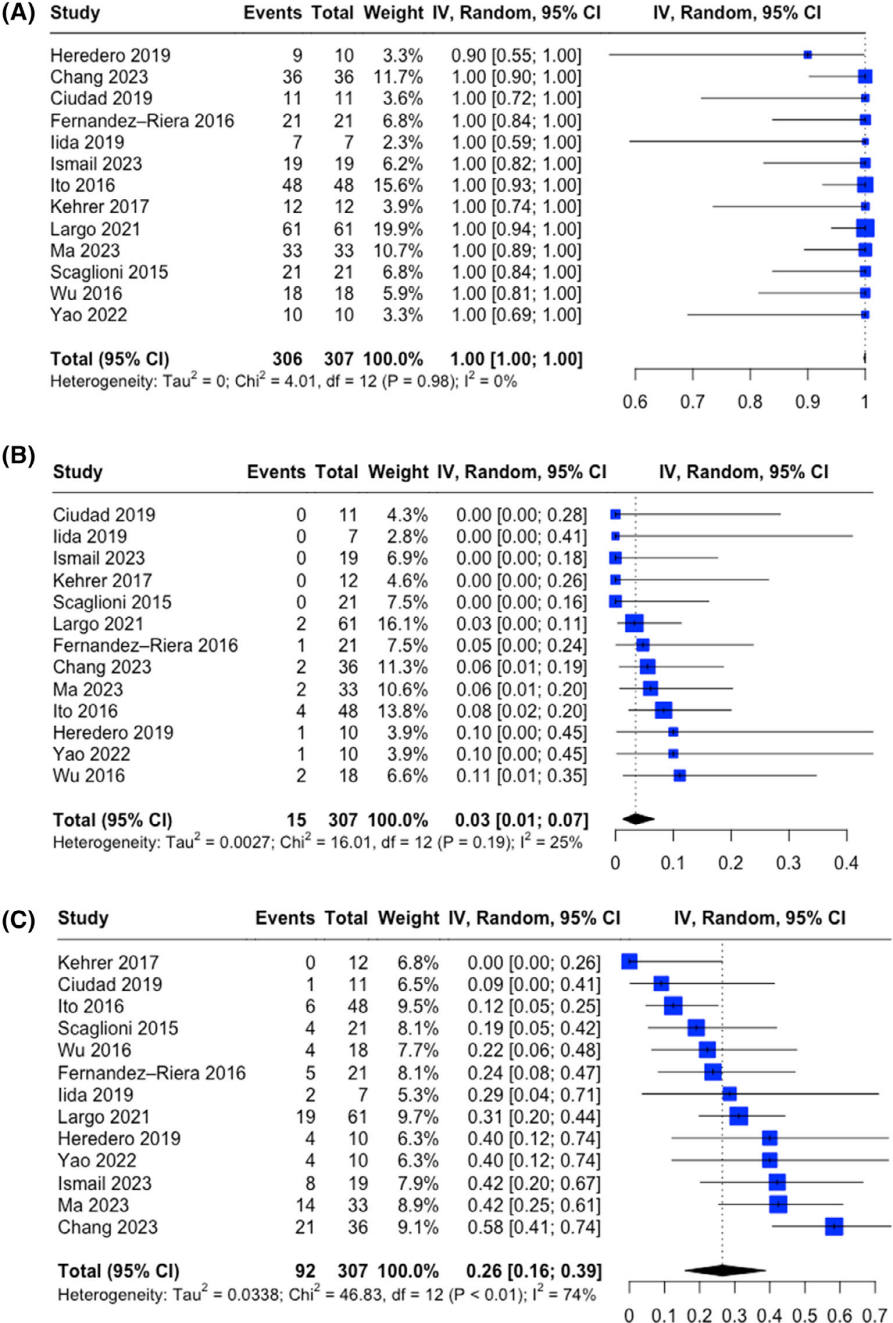
Forest plots showing the pooled (A) flap survival rate, (B) revision rate for major complication, and (C) overall complication rate. The dashed vertical line represents the overall measure of effect. [Color figure can be viewed at wileyonlinelibrary.com]

A single arm meta‐analysis was performed for flap survival rate, reoperation rate for major complication, and overall complication rate. Arcsine transformation of the data was carried out for the analysis of overall proportion.^17^ Inverse variance method (DerSirmonian–Laird estimator) was used to estimate the between‐study variance (*τ* ^2^).^18^ Results are presented as pooled estimates with 95% CIs. A forest plot graph was created for each outcome (Figure 1).

Cochran's *Q* method was used to assess between studies heterogeneity.^19^ *I* ^2^ was calculated as a measure of heterogeneity. The *I* ^2^ value represents the percentage of total variation across studies caused by heterogeneity rather than by chance.^20^ According to the Cochrane criteria, values from 0% to 40% may signify low heterogeneity, 30% to 60% may represent moderate heterogeneity, 50% to 90% may represent substantial heterogeneity, and 75% to 100% represents considerable heterogeneity. In order to have a more conservative approach, we considered the variability between surgeons and patients within each included studies and we used a random effects model for all comparisons. Influence analysis^21^ was performed to identify potential influential studies for the abovementioned outcomes. In particular, a leave‐one‐out meta‐analysis was performed to test the influence of each included study on the overall effect and *I* ^2^ heterogeneity. Scatter plots (Bajuat plot) were created for each outcome.^22^ The horizontal axis (x‐axis) shows the contribution of each study to the overall *Q* test statistic for heterogeneity. The standardized difference of the overall treatment effect is plotted on the vertical axis, showing the influence of each study on the pooled effect size.

Analysis of publication bias was performed by visual inspection of the funnel plot and using the Egger's regression test,^23^ which statistically examines the asymmetry of the funnel plot.

All the analyses were performed using the R software for statistical computing (R version 4.2.2; “meta” and “dmetar” packages). Statistical significance was defined as *p* < 0.05.^24^

## 3 RESULTS

### 3.1 Literature search results and study description

**FIGURE 2:**
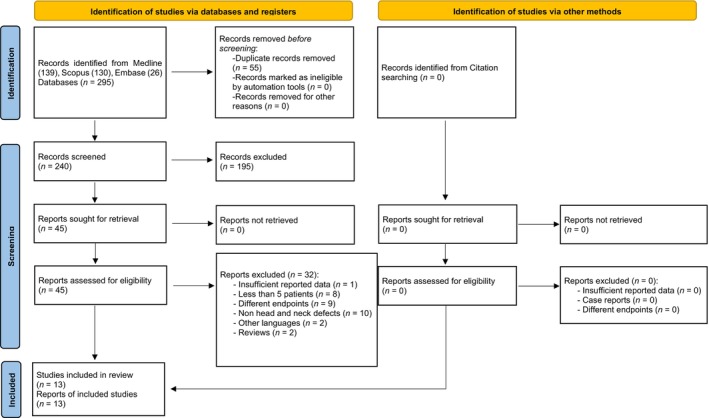
PRISMA flow diagram. [Color figure can be viewed at wileyonlinelibrary.com]

Figure 2 shows the flowchart of the study identification process. The search strategy retrieved a total of 295 potentially relevant publications, of which 13 were finally included.^1^, ^5^, ^12^, ^25^, ^26^, ^27^, ^28^, ^29^, ^30^, ^31^, ^32^, ^33^, ^34^

Studies' characteristics are summarized in Table 1. A total of 305 patients (males: 80.8%, *n* = 232/281) with a median age of 56.1 years (*n* = 305/305; 95% CI 53.9–63) who underwent a total of 307 PAP flap reconstruction of head and neck defects. Patient's BMI ranged from 16 to 41.6, with a median value of 24.0 (*n* = 238/305; 95% CI 23.6–25.4). The oral cavity soft tissue defects stood out as predominant (*n* = 150/252, 59.5%), with head and neck skin defects following in frequency (*n* = 72/252, 28.6%). However, this flap was used also in reconstructing oral cavity defects involving bony structures such as the mandible or the maxilla (*n* = 17/252, 6.8%), and for pharyngo‐laryngo‐esophageal defects (*n* = 11/252, 4.4%). Cancer accounted for 94.5% of the cases requiring reconstruction (*n* = 290/307), while other indications for surgery included neck contractures (*n* = 7/252, 2.8%), plate exposure (*n* = 3/252, 1.2%), intraoral fistula (*n* = 3/252, 1.2%), osteoradionecrosis (*n* = 2/252, 0.8%), and prior failed flap (*n* = 2/252, 0.8%).

**TABLE 1:** Studies' general characteristics.

Author	Country of origin	No. of patients (males)	Age (range)	BMI (range)	No. of flaps	Site of defects	Flap survival rate	Partial necrosis	Reoperation rate for major complication	Overall complication rate	Surgical indication
Chang et al.^1^	USA	35 (29)	65.8 (NA)	24.0 (NA)	36	2 OC, 27 H&N skin, 4 mandible or maxilla, 3 other	100%, *n* = 36	0%, *n* = 0	5.6%, *n* = 2 (hematoma, venous thrombosis)	58.3%, *n* = 21	36 cancer
Ciudad et al.^26^	Taiwan	11 (8)	59.5 (54–63)	NA	11	6 H&N skin, 1 mandible or maxilla, 4 other	100%, *n* = 11	0%, *n* = 0	0%, *n* = 0	9.1%, *n* = 1	5 cancer; 6 neck contracture
Fernandez‐Riera et al.^27^	Taiwan	21 (19)	53.9 (25–86)	24.78 (20.1–32.3)	21	21 OC	100%, *n* = 21	0%, *n* = 0	4.8%, *n* = 1 (hematoma)	23.8%, *n* = 5	21 cancer
Heredero et al.^28^	Spain	10 (6)	53.9 (28–71)	27.8 (20.4–37.3)	10	10 OC	90%, *n* = 9	0%, *n* = 0	10%, *n* = 1 (flap failure)	40%, *n* = 4	10 cancer
Iida et al.^29^	Japan	7 (3)	70.3 (59–83)	NA	7	4 OC, 2 PLE, 1 mandible or maxilla	100%, *n* = 7	0%, *n* = 0	0%, *n* = 0	28.6%, *n* = 2	7 cancer
Ismail et al.^30^	USA	19 (NA)	61.29 (NA)	22.65 (NA)	19	19 OC	100%, *n* = 19	5.2%, *n* = 1	0%, *n* = 0	42.1%, *n* = 8	19 cancer
Ito et al.^12^	Taiwan	48 (41)	53.3 (32–77)	23.6 (17.2–41.6)	48	NA	100%, *n* = 48	6.25%, *n* = 3	8.3%, *n* = 4 (NOS)	12.5%, *n* = 6	48 cancer
Kehrer et al.^31^	Taiwan	12 (12)	53.5 (29–67)	25.6 (19.31–30.7)	12	12 OC	100%, *n* = 12	0%, *n* = 0	0%, *n* = 0	0%, *n* = 0	12 cancer
Largo et al.^32^	USA	60 (46)	63 (18–90)	24 (16–36)	61	21 OC, 27 H&N skin, 4 PLE, 8 mandible or maxilla, 1 other	100%, *n* = 61	3.3%, *n* = 2	3.3%, *n* = 2 (partial flap necrosis)	31.2%, *n* = 19	54 cancer; 3 intraoral fistula; 2 prior failed flap; 2 osteoradionecrosis
Ma et al.^33^	China	33 (26)	54.2 (30–74)	25.4 (20.2–30)	33	31 OC, 1 PLE, 1 other	100%, *n* = 33	3.0%, *n* = 1	6.1%, *n* = 2 (venous thrombosis)	42.4%, *n* = 14	33 cancer
Scaglioni et al.^34^	Taiwan	21 (19)	56.1 (36–79)	NA	21	16 OC, 3 PLE, 2 H&N skin	100%, *n* = 21	0%, *n* = 0	0%, *n* = 0	19%, *n* = 4	20 cancer; 1 neck contracture
Wu et al.^5^	Taiwan	18 (16)	55.7 (41–76)	NA	18	14 OC, 1 PLE, 3 mandible or maxilla	100%, *n* = 18	5.5%, *n* = 1	11.1%, *n* = 2 (NOS)	22.2%, *n* = 4	15 cancer; 3 plate exposures
Yao et al.^25^	USA	10 (6)	71.3 (52–83)	NA	10	10 H&N skin	100%, *n* = 10	0%, *n* = 0	10%, *n* = 1 (hematoma)	40%, *n* = 4	10 cancer

Abbreviations: H&N, head and neck; OC, oral cavity; PLE, pharyngo‐laryngo‐esophageal.

### 3.2 Methodological quality of included studies

Table 2 summarizes the risk of bias of the included studies using the NICE quality assessment tool. The risk of bias assessment showed a generally moderate quality of included studies, with a median score of 6. The main limitation is that only two studies (*n* = 19/307, 6.2%) reported a prospective data acquisition, and that no multicentric study was included. Finally, 12 studies (*n* = 297/307, 96.7%) reported a consecutive patient enrollment, reducing the risk of selection bias.

**TABLE 2:** Quality assessment of case series studies checklist from National Institute for Health and Clinical Excellence: (1) Was the case series collected in more than one center (i.e., multi‐center study?) (2) Is the hypothesis/aim/objective of the study clearly described? (3) Are the inclusion and exclusion criteria (case definition) clearly reported? (4) Is there a clear definition of the outcomes reported? (5) Were data collected prospectively? (6) Is there an explicit statement that patients were recruited consecutively? (7) Are the main findings of the study clearly described? (8) Are outcomes stratified (e.g., by abnormal results, disease stage, patient characteristics)?

Author	1	2	3	4	5	6	7	8	Total
Chang et al.^1^	No	Yes	Yes	Yes	No	Yes	Yes	Yes	6
Ciudad et al.^26^	No	Yes	Yes	Yes	No	Yes	Yes	Yes	6
Fernàndez‐Riera et al.^27^	No	Yes	Yes	Yes	No	Yes	Yes	Yes	6
Heredero et al.^28^	No	Yes	Yes	Yes	No	Yes	Yes	Yes	6
Iida et al.^29^	No	Yes	Yes	Yes	Yes	Yes	Yes	Yes	7
Ismail et al.^30^	No	Yes	Yes	Yes	No	Yes	Yes	Yes	6
Ito et al.^12^	No	Yes	Yes	Yes	No	Yes	Yes	Yes	6
Kehrer et al.^31^	No	Yes	Yes	Yes	Yes	Yes	Yes	No	6
Largo et al.^32^	No	Yes	Yes	Yes	No	Yes	Yes	Yes	6
Ma et al.^33^	No	Yes	Yes	Yes	No	Yes	Yes	Yes	6
Scaglioni et al.^34^	No	Yes	Yes	Yes	No	Yes	Yes	No	5
Wu et al.^5^	No	Yes	Yes	Yes	No	Yes	Yes	Yes	6
Yao et al.^25^	No	Yes	Yes	Yes	No	No	Yes	No	4

**FIGURE 3:**
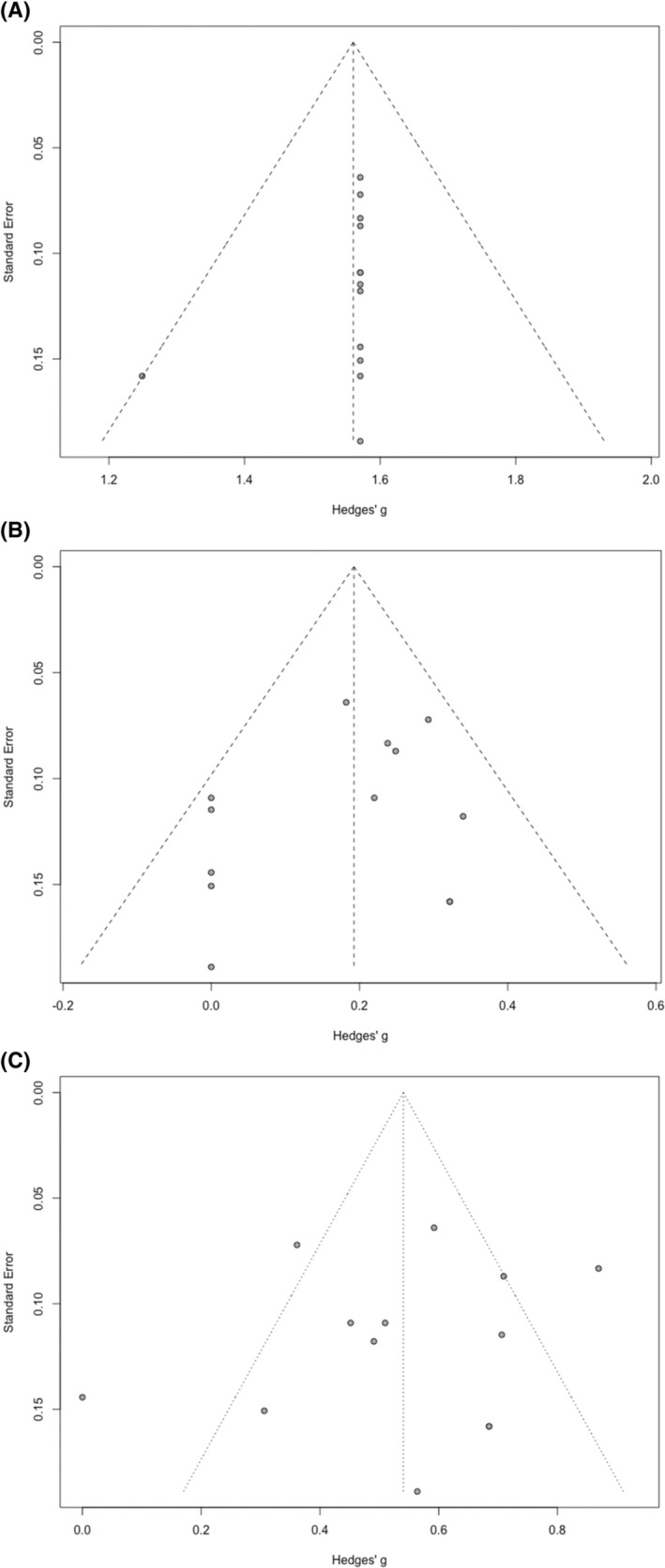
Funnel plots for evaluation of publication bias using the effect size for (A) flap survival rate, (B) revision rate for major complication, and (C) overall complication rate.

The funnel plots generated for the meta‐analysis of each intervention are shown in Figure 3. Egger's regression test did not indicate the presence of distribution asymmetry in the points of all funnel plots (flap survival rate, Intercept = 0.61, *p* = 0.250, Figure 3A; reoperation rate for major complication, Intercept = 1.36, *p* = 0.236, Figure 3B; overall complication rate, Intercept = 1.13, *p* = 0.543, Figure 3C), suggesting no obvious publication bias.

### 3.3 Flap characteristics

The median length of the PAP flap was 12.46 cm (*n* = 224/307; 95% CI 11.5–19.9), with a range of 5–32 cm (*n* = 210/307, 68.4%), while the median width was 7.4 cm (*n* = 242/307; 95% CI 6.6–7.7), ranging from 4 to 25 cm (*n* = 228/307, 74.3%). The overall median surface area measured 106.7 cm^2^ (*n* = 261/307; 95% CI 81.0–153.3), varying from 20 to 320 cm^2^ (*n* = 209/307, 68.1%). Flap thickness ranged from 0.5 to 4 cm, but it was reported in only two included studies (*n* = 79/307, 25.7%). The number of profunda artery perforators varied from 1 to 5 perforators, with a median of two perforators per flap (*n* = 74/307; 95% CI 1.4–2.4). The pedicle measured between 6 and 15 cm (*n* = 177/307, 57.7%), with a median length of 9.8 cm (*n* = 264/307; 95% CI 8.4–10.2). Median arterial and venous diameter measured 2.1 mm (*n* = 164/307, 95% CI 1.6–2.2) and 2.4 mm (*n* = 206/307, 95% CI 2.1–2.9), respectively, with the artery ranging from 0.3 to 2.5 mm (*n* = 115/307, 37.5%) and the vein from 0.8 to 4 mm (*n* = 126/307, 41%) (Table 3, (Figure S1, Supporting Information)).

**TABLE 3:** Flaps characteristics.

Author	No. of flaps	Flap length (range)	Flap width (range)	Flap surface area (range)	Flap thickness (range)	No. of perforators (range)	Arterial diameter (range)	Venous diameter (range)	Pedicle length (range)
Chang et al.^1^	36	NA	NA	NA	NA	NA	2.2 (NA)	2.9 (NA)	10.1 (NA)
Ciudad et al.^26^	11	27.7 (25–30)	7.6 (6–9)	212.3 (156–261)	NA	1.4 (1–2)	NA	NA	8.6 (7–10)
Fernandez‐Riera et al.^27^	21	6.4 (5–8)	10 (6–16)	68.1 (30–128)	NA	NA	2.02 (1.5–2.5)	2.3 (1.6–4)	8.4 (6.5–10.5)
Heredero et al.^28^	10	11.5 (8–15)	7.4 (6–10)	87.3 (48–135)	1 (0.4–3.5)	NA	1.57 (1–2.5)	1.76 (0.75–4)	7.3 (6–9)
Iida et al.^29^	7	18.1 (15–22)	7.85 (6–14)	147 (102–308)	NA	NA	1.72 (1.2–2.5)	2.46 (1–3)	8.28 (6–11)
Ismail et al.^30^	19	NA (10–16)	NA (7–10)	106.7 (NA)	NA	NA	NA	3.2 (NA)	8.1 (NA)
Ito et al.^12^	48	19.9 (8.5–32)	7.7 (6–10)	153.2 (48–320)	NA	2.0 (1–3)	NA	NA	9.8 (7–13)
Kehrer et al.^31^	12	10.9 (7–26)	10.9 (7–25)	120 (77–221)	NA	2.4 (1–5)	0.8 (0.3–1.2)	NA	NA
Largo et al.^32^	61	12.1 (5–24)	7.1 (4–14)	92.2 (20–240)	1.9 (0.5–4)	NA	NA	NA	11.5 (8–15)
Ma et al.^33^	33	12.5 (NA)	6.4 (NA)	79.7 (NA)	NA	NA	1.39 (1–2)	1.9 (1.5–2.5)	8.4 (NA)
Scaglioni et al.^34^	21	16.8 (12–27)	6.6 (5–8)	113 (72–189)	NA	1.6 (1–3)	2.13 (1.8–2.4)	2.78 (2–4)	10.2 (8–13)
Wu et al.^5^	18	NA	7.7 (6–9)	166.1 (90–243)	NA (0.5–2)	2 (1–3)	2–2.5	NA	9.8 (8–13)
Yao et al.^25^	10	NA	NA	NA	NA	NA	NA	2.4 (2–4)	NA

### 3.4 Surgical treatment and complications

**FIGURE 4:**
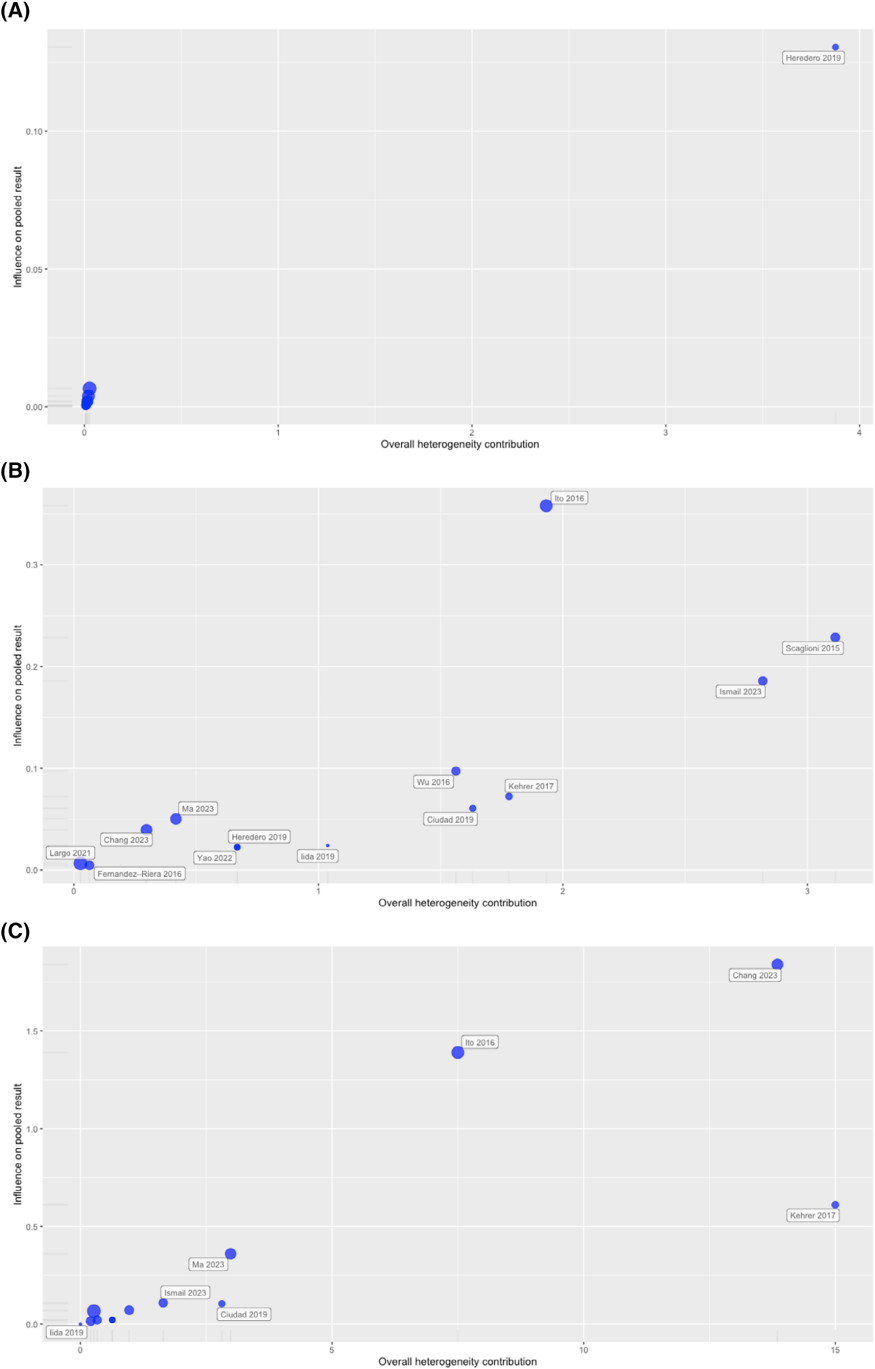
Baujat plots showing the studies contribution to the overall heterogeneity for (A) flap survival rate, (B) revision rate for major complication, and (C) overall complication rate. [Color figure can be viewed at wileyonlinelibrary.com]

Surgical outcomes of individual studies are shown in Table 1. A random effect modeling showed a pooled flap survival rate of 100% (*n* = 306/307; 95% CI 99.8%–100%; Figure 1A). No significant heterogeneity was measured between studies (*Q* = 4.01, *p* = 0.98). In particular, the between‐study variance was estimated at *τ* ^2^ = 0 (95% CI 0–0), with an *I* ^2^ value of 0% (95% CI 0%–56.6%). Baujat plot showing the studies contribution to the overall heterogeneity is shown in Figure 4A. The pooled reoperation rate for major complication measured using a random effect modeling was 3.5% (*n* = 15/307; 95% CI 1.25%–6.8%; Figure 1B), with moderate between‐study heterogeneity (*Q* = 16.01, *p* = 0.191). The between‐study variance was estimated at *τ* ^2^ = 0.003 (95% CI 0.000–0.383), with an *I* ^2^ value of 25.1% (95% CI 0%–61.1%). Baujat plot showing the studies contribution to the overall heterogeneity is shown in Figure 4B. The pooled overall complication rate measured with a random effect modeling was 26.5% (*n* = 92/307; 95% CI 15.7%–38.9%; Figure 1C), with significant heterogeneity (*Q* = 46.8, *p* < 0.05). The between‐study variance was estimated at *τ* ^2^ = 0.034 (95% CI 0.011–0.120), with an *I* ^2^ value of 74.4% (95% CI 55.7%–85.2%). Baujat plot showing the studies contribution to the overall heterogeneity is shown in Figure 4C. Influence analysis identified no influential studies on the cumulative outcomes rate.

Postoperative complications were reported in 100% of the included studies (*n* = 307/307), with the most common being wound dehiscence (*n* = 15/307, 4.9%), delayed healing (*n* = 14/307, 4.6%), and wound infection (*n* = 12/307, 3.9%). Partial flap necrosis and hematoma occurred in 2.6% of cases (*n* = 8/307), while arterial and venous thrombosis were documented in 0.7% (*n* = 2/307) and 1.3% (*n* = 4/307), respectively.

## 4 DISCUSSION

This study represents the largest cohort of PAP flaps performed for head and neck reconstruction, showing optimistic surgical outcomes with a pooled flap survival rate of 100%, and a pooled reoperation rate for major complication of only 3.5% (*n* = 15/307). From an anatomical point of view, PAP flap confirmed its inherent versatility, which enabled it to accurately reconstruct a wide range of head and neck defects. The harvested surface area ranged from 20 to 320 cm^2^, demonstrating an impressive range of dimensions, with lengths varying from 5 to 32 cm and widths from 4 to 25 cm. This remarkable variability enables the reconstruction of a wide spectrum of defects, from small oral cavity defects to extensive skin defects in the head and neck region. Moreover, the PAP flap has been successfully employed in the reconstruction of tubulized pharyngo‐laryngo‐esophageal defects, further highlighting its versatility and effectiveness in addressing diverse anatomical challenges.

**FIGURE 5:**
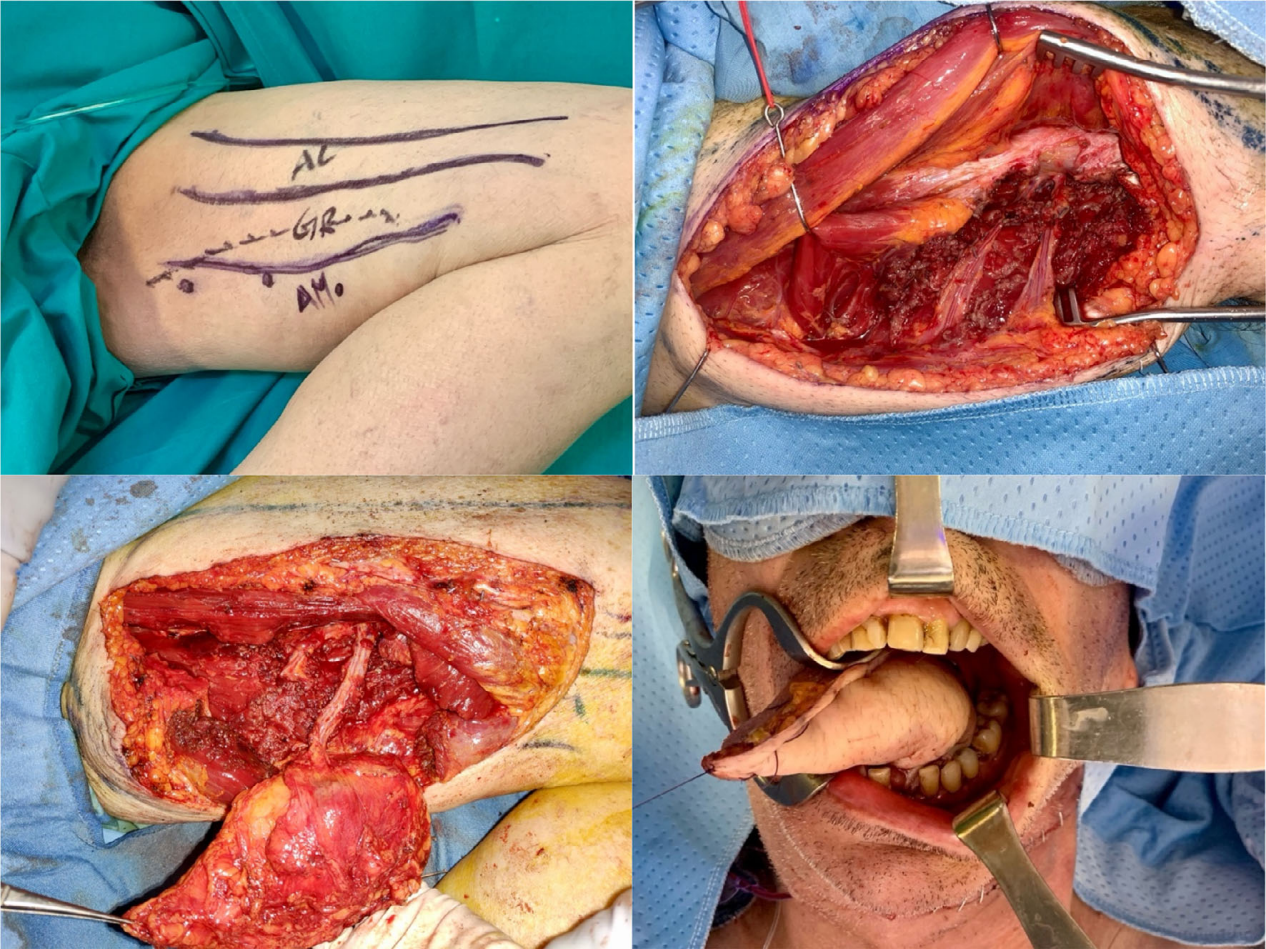
(top left) Preoperative design of PAP flap. Positioning the thigh in abduction and the knee flexed to 90°. The margins of the adductor longus (AL), gracilis (G), and adductor magnus (AM) muscles are drawn. (top right and bottom left) Flap harvest. (bottom right) Glossectomy reconstruction. [Color figure can be viewed at wileyonlinelibrary.com]

Perforator free flaps have become increasingly popular in recent decades for head and neck reconstruction, despite their challenging harvesting process.^35^, ^36^ The inclusion of perforating vessels provides excellent freedom of movement, as the design of free flaps is solely based on the perforator within the flap^37^ (Figure 5). The ideal perforator flap offers a predictable blood supply, sufficient pedicle length, and minimal functional morbidity, leading to improved cosmetic outcomes. While numerous reconstructive options exist for head and neck defects, surgeons and researchers are dedicated to exploring alternatives such as the PAP flap, which is demonstrating promising advantages over other commonly used perforator free flaps.

As demonstrated by the wide variability of application reported by the included articles, the median vascular pedicle length of 9.8 cm facilitated the use of PAP flap in many reconstructive sites, reaching with ease major cervical vessels for anastomosis. The oral cavity emerged as the primary site for the application of this flap (Figure 5). Both Fernandez‐Riera et al. and Heradero et al. emphasized its pliability, attributed to the thinner and softer dermis of the inner thigh, which facilitated smoother insetting for reconstructing defects affecting the tongue and oral floor.^27^, ^28^ Several articles in the literature, including Ito et al. and Scaglioni et al., have positively emphasized the consistency of perforators and their uniform trajectory, which follows a straight course, thereby facilitating dissection.^12^, ^34^ The maximum number of perforators identified and preserved during flap harvesting is 5, with a median of 2 per flap.^31^ The flap thickness was found to be a highly variable feature, strongly linked to patients' BMI, ranging from 0.4 to 4 cm. Nevertheless, for thicker flaps, thinning procedures, though sometimes laborious, are effective. On the other hand, in situations requiring additional tissue, PAP flap can be combined with the adductor magnus muscle or with the transverse upper gracilis (TUG) flap to enhance tissue volume and reliability.^26^, ^30^ Despite the advantages, several authors pointed out that the conditions for a chimeric flap are not always met. Heredero et al. reported an adequate vascular configuration for a chimeric flap in less than 30% of the studied population using angiography.^28^ Additionally, Yao et al. were only successful in performing a chimeric flap in 3 out of 10 cases within the patient cohort. This necessitated the harvest of two separate flaps and, consequently, required two independent microvascular anastomoses.^25^ Although the mean operating time is reported too sporadically to conduct significant statistical analyses, the anatomical location of the donor site permits simultaneous surgery by two teams, adopting a supine frog‐leg position, without significantly affecting the total duration of the procedure.^5^, ^12^, ^28^, ^32^, ^34^ Additionally, the skin on the posteromedial thigh demonstrates a greater degree of laxity compared to that of the anterolateral thigh. This simplifies achieving primary closure of the donor site, eliminating the need for time‐consuming graft harvesting and placement.

The pooled overall complication rate was determined to be 26.5%. Among these, the most frequently encountered complications were wound dehiscence (4.9%), delayed healing (4.6%), and wound infection (3.9%). Partial flap necrosis and hematoma occurred in 2.6% of cases, while arterial and venous thrombosis were documented in 0.7% and 1.3%, respectively. It is important to consider that a significant portion of the study population had undergone multimodality adjuvant therapies, were smokers, had previous surgeries, or required multiple free flaps for defect reconstruction. It should be noted that these surgical complications were primarily transient in nature and that only 3.5% of patients needed reoperation because of a mayor complication. In this context, Cho et al. have identified high BMI, older age, and a history of smoking as risk factors for the development of donor site complications in a population undergoing breast reconstruction with PAP flap.^9^

Our analysis showed that this flap was selected for patients with a median age of 56.1 years. The PAP flap represents a suitable choice for younger patients, who are more attentive to the aesthetic result besides the oncological and functional outcomes. Specifically, the scar's less visible position compared to the ALT flap tends to be more satisfactory for patients.^38^ Moreover, the usually less dense hair distribution in the thigh's medial region is often perceived as an advantage at the level of the reconstructed site. Additionally, no patients reported permanent restriction or decreased leg adduction. This may be attributed to the fact that leg adduction is governed by five muscles and that unroofing of perforators, especially muscle‐septal perforators, results in a more direct trajectory. This facilitates minimizing the need for sectioning the adductor magnus muscle and promotes ease in repair through suturing.

The outcomes we reported for the reconstruction of head and neck defects using PAP flaps are comparable to those observed in other reconstruction subsites. Specifically, the literature provides more extensive data on breast reconstruction. Both Atzeni et al.^39^ and Jo et al.^40^ confirmed the reliability of this flap, reporting zero flap losses with their series of 116 and 43 PAP flaps, respectively. Additionally, both studies noted similar complication rates, with donor site wound dehiscence being the most common complication. Cho et al., in his study of 130 PAP flaps, also identified wound dehiscence as a frequent issue, and found it to be correlated with the body mass index of the patients.^9^

This study is subject to several limitations. Nearly all the included studies were single‐center retrospective cohorts with small sample sizes and thus prone to several biases. Moreover, it was not possible to perform any stratified analysis based on the reconstructed site nor on patient's previous treatments. Additionally, given the variability in the site of head and neck defects, conducting assessments of functional outcomes at the reconstructed site was not feasible. Also, the definition of complications may vary between the included studies, and the relative high heterogeneity of the pooled estimates should be taken into account when analyzing our results. Finally, a direct comparison between the PAP and other flaps was not conducted based on the absence of available literature data. Therefore, further multicentric studies are needed to better define the indications and outcomes of PAP flap in head and neck reconstruction.

## 5 CONCLUSIONS

Our systematic review and meta‐analysis showed that the application of the PAP flap is a valid and versatile option in head and neck reconstructions. This flap showed several favorable aspects, such as an exceptionally low flap failure rate, versatility in achieving variable dimensions and a relatively low incidence of major complications. PAP flap might be considered as a compelling alternative to the traditionally employed soft tissue free flaps in head and neck reconstruction.

## CONFLICT OF INTEREST STATEMENT

The authors declare no conflicts of interest.

## ETHICS STATEMENT

Ethics approval was not required for this study since all reported data were obtained from the available published literature and all patients were deidentified. Informed consent was not required for this study since all reported data were obtained from the available published literature and all patients were deidentified.

## Supporting information


**Figure S1.** Forest plots showing the pooled (A) PAP flap surface area, (B) artery diameter, (C) vein diameter.

## Data Availability

The data that support the findings of this study are available on request from the corresponding author. The data are not publicly available due to privacy or ethical restrictions.
