# Supplementary material for: Surgical outcomes of profunda artery perforator flap in head and neck reconstruction: A systematic review and meta‐analysis
Source: Head Neck. 2024 Jul 30;47(1):98–111. doi: 10.1002/hed.27891 (PMC11635749; doi:10.1002/hed.27891)
Supplement: Supplementary file 1 — Figure S1. Forest plots showing the pooled (A) PAP flap surface area, (B) artery diameter, (C) vein diameter. [file HED-47-98-s001.docx]

**Supplementary Material legends**

Supplementary Material: Forest plots showing the pooled (A) PAP flap surface area, (B) artery diameter, (C) vein diameter.

**Supplementary Material**

**A)**

**
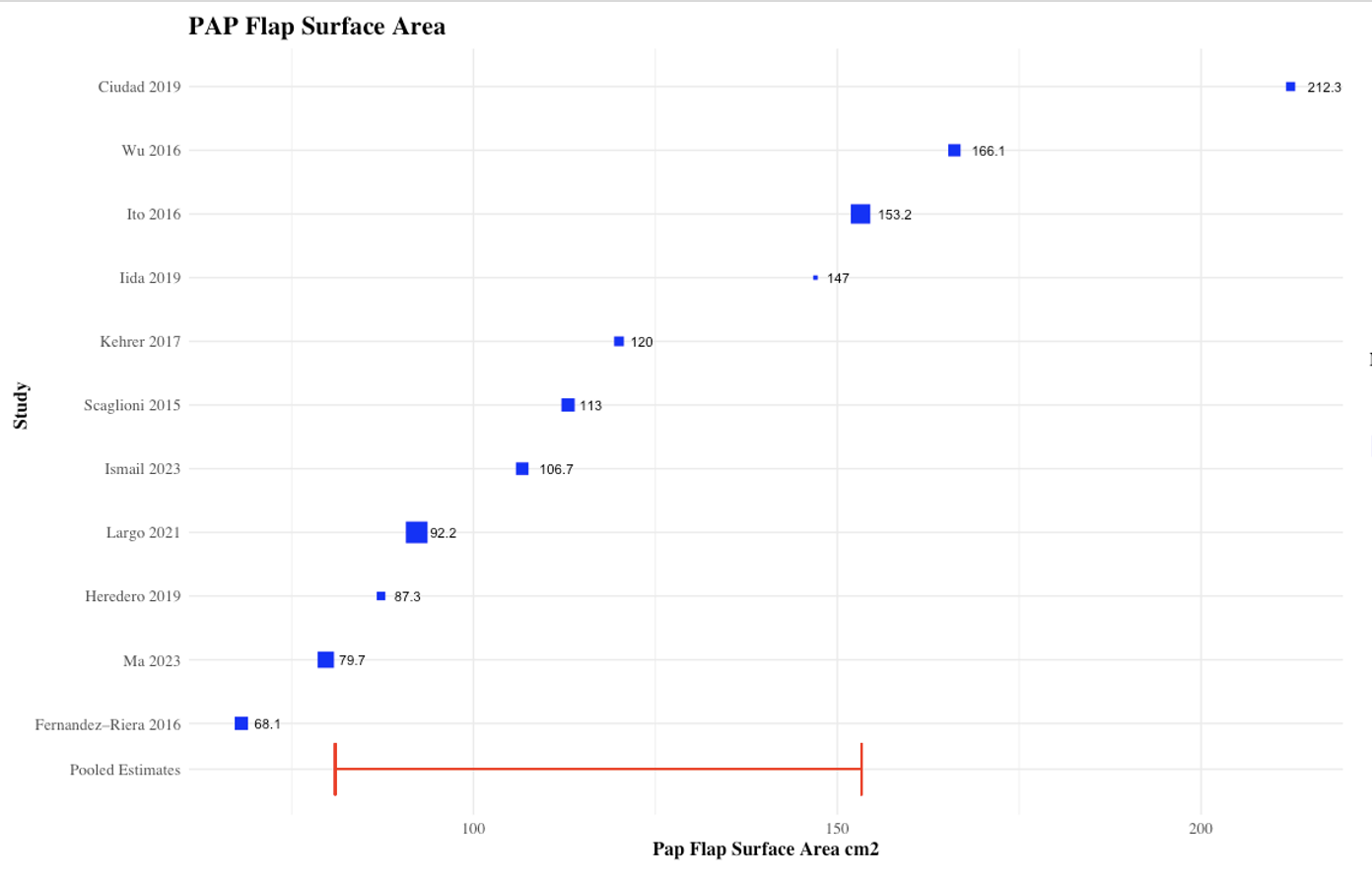
**

**B)
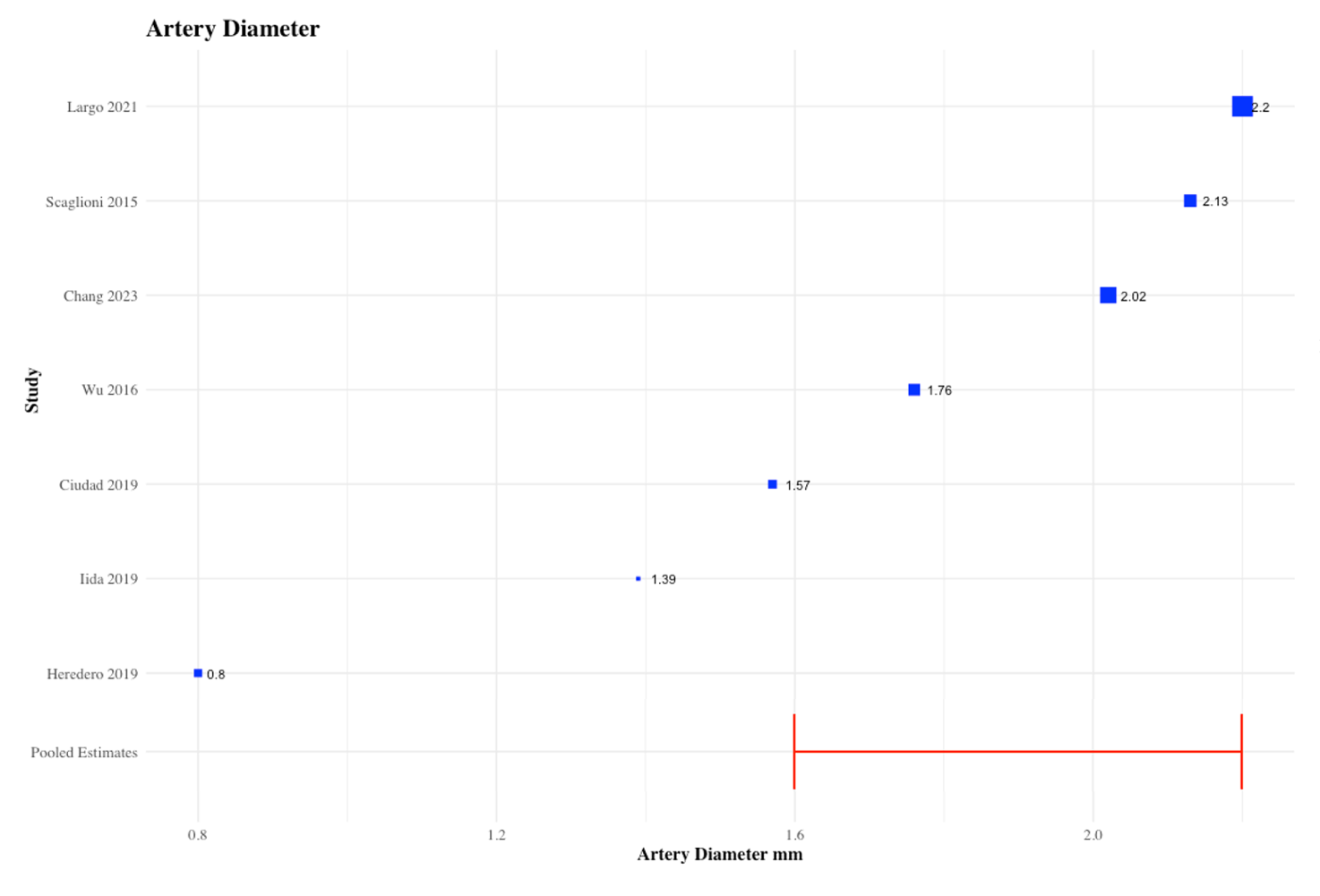
**

**C)
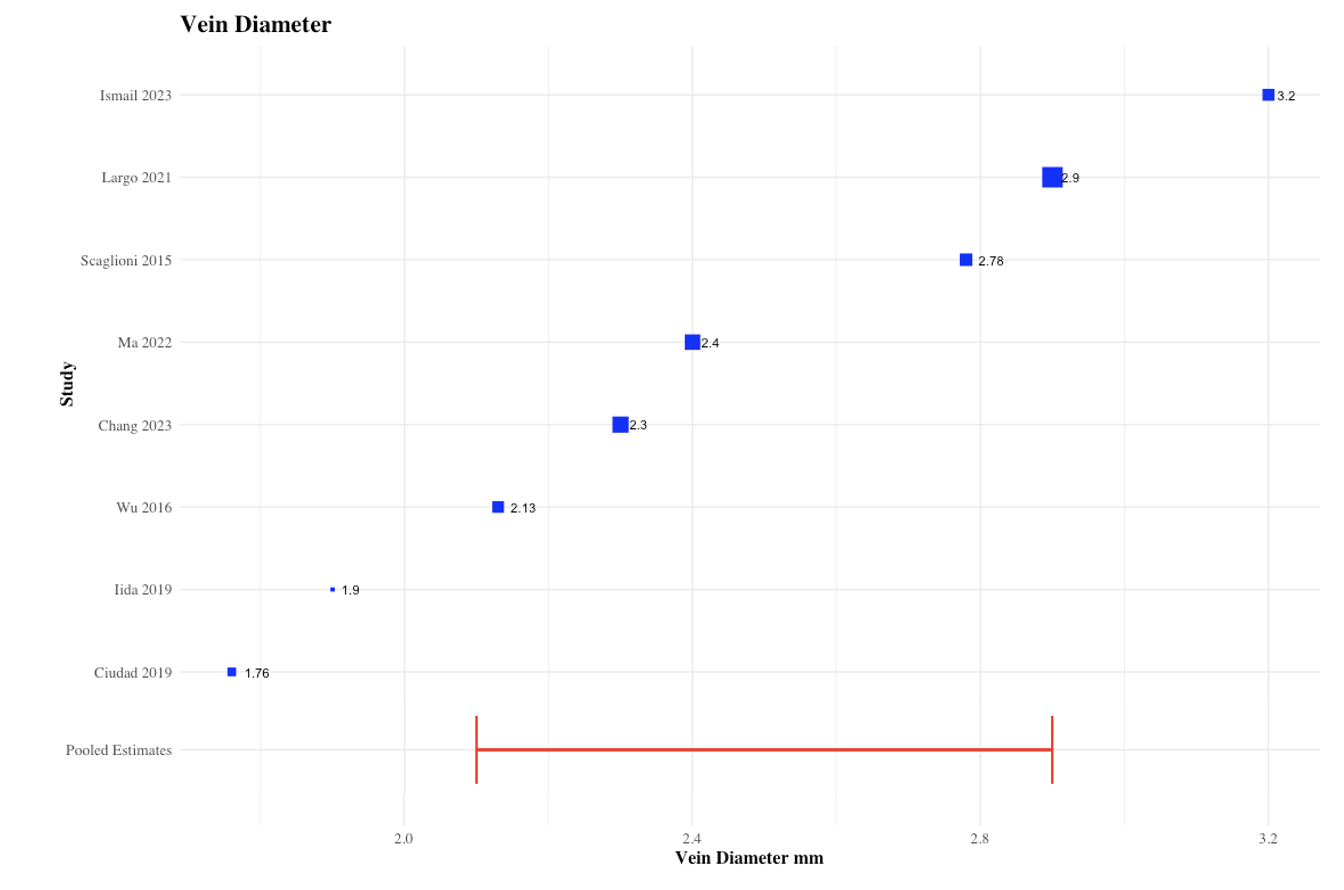
**
